# Association of brain cortical changes with efficacy of treatment in patients with chronic neck and shoulder pain: a longitudinal surface-based morphometry study

**DOI:** 10.3389/fnins.2026.1738646

**Published:** 2026-03-03

**Authors:** Zhiqiang Qiu, Jinming Tong, Maojiang Yang, Libing He, Hongjian Li, Tianci Liu, Xiaoxue Xu

**Affiliations:** 1 Department of Radiology, Affiliated Hospital of North Sichuan Medical College, Nanchong, China; 2 Department of Pain, Affiliated Hospital of North Sichuan Medical College, Nanchong, China

**Keywords:** chronic neck and shoulder pain, cortical morphological, longitudinal study, minimally invasive interventional treatment, MRI

## Abstract

**Objective:**

Studies have shown that the pathophysiological mechanisms of Chronic neck and shoulder pain (CNSP) involve not only local spinal and neural abnormalities but also abnormal brain cortical structures related to pain modulation. However, it remains unclear how these cortical alterations may influence efficacy of treatment.

**Materials and methods:**

31 CNSP patients and 30 age- and gender-matched healthy controls (HCs) underwent 3D high-resolution structural magnetic resonance imaging (MRI) scans. The CNSP patients underwent a second MRI scan 3 months after receiving minimally invasive interventional treatment. The longitudinal changes in cortical thickness (CT), fractal dimension (FD), gyrification index (GI), and sulcal depth (SD) were studied before and after treatment in the CNSP patients, and conducted partial correlation analysis with treatment efficacy.

**Results:**

Compared to healthy controls, CNSP patients at baseline exhibited significant reduced cortical thickness (CT) in the bilateral precentral gyrus, superior frontal gyrus, lingual gyrus, left paracentral lobule, fusiform gyrus, superior temporal gyrus, supramarginal gyrus, and right precuneus. Deeper sulcal depth (SD) was observed in the bilateral central sulcus, anterior and posterior cingulate cortices, insula, lateral orbitofrontal cortex (OFC), and left dorsolateral prefrontal cortex (DLPFC). Additionally, an increased Gyrification Index (GI) was found in the bilateral lingual gyrus, left lateral OFC, anterior/posterior cingulate cortices, and right medial OFC. Three months after minimally invasive intervention, these morphological abnormalities showed widespread normalization. Correlation analyses revealed that higher baseline CT in the left precentral gyrus and paracentral lobule, lower baseline SD in the left cingulate cortex and central sulcus, and higher baseline GI in the right medial OFC were significant predictors of greater pain relief. Furthermore, the longitudinal restoration of CT in the left precentral gyrus and SD normalization in the left DLPFC and cingulate cortex were positively correlated with the reduction in VAS scores.

**Conclusion:**

This study identifies specific morphological alterations characterized by cortical thinning and increased sulcal depth in the sensorimotor cortex (precentral gyrus, paracentral lobule, central sulcus) and the pain modulation network (cingulate cortex, DLPFC, OFC) as key biomarkers for CNSP. The findings demonstrate that baseline structural integrity in these specific regions serves as a robust predictor of treatment efficacy. Moreover, the longitudinal structural recovery paralleling pain relief confirms the reversible nature of maladaptive neuroplasticity, highlighting CT in the precentral gyrus and SD in the DLPFC as critical indicators for evaluating chronic pain interventions.

## 1 Introduction

Chronic pain in the neck and shoulders (CNSP) is a common musculoskeletal condition that greatly affects the well-being and work performance of those who suffer from it. It is characterized by persistent discomfort in the cervical and shoulder regions lasting for more than 3 months (Cohen, 2015). The incidence of CNSP is notably high, with a systematic review reporting global annual and lifetime prevalence rates of 37.2 and 48.5%, respectively (Fejer et al., 2006). A recent analysis of healthcare costs in the U.S. revealed that low back and neck pain contribute the highest medical expenditures among 154 diseases, with an estimated total of $134.5 billion (Dieleman et al., 2020; Murray et al., 2013).

While the pathology of CNSP involves local musculoskeletal dysfunction, growing evidence implicates maladaptive neuroplasticity within the central nervous system. Previous neuroimaging studies have established a link between CNSP and structural alterations in the cerebral cortex, distinguishing patients from healthy controls (HCs) (De Pauw et al., 2019; Niddam et al., 2019). For instance, cortical morphology appears to vary by etiology: reduced volume in the precentral and superior temporal gyri has been observed in chronic whiplash-associated neck pain, whereas cortical thickening in the superior parietal lobule has been reported in idiopathic neck pain (De Pauw et al., 2019). Similarly, surface-based analyses in chronic shoulder pain have revealed reduced sulcal depth in the sensorimotor and insular. Crucially, these structural anomalies are not merely epiphenomena but are clinically relevant; cortical thickness and sulcal depth in these regions have been shown to correlate significantly with motor deficits, pain intensity, and pain-related emotional dysregulation (Niddam et al., 2019). These findings suggest that CNSP is associated with a disruption in the brain’s structural integrity, potentially affecting networks involved in nociception and sensorimotor integration.

Although the cross-sectional association between brain structural abnormalities and chronic pain is well-documented, the longitudinal trajectory of these changes following clinical intervention involves complex dynamics that remain under-explored in CNSP. Prior longitudinal research in chronic low back pain (CLBP) provides compelling evidence that structural deficits are not permanent but can be reversible. Specifically, Seminowicz et al. (2011) demonstrated that effective pain relief following surgery or injections resulted in increased cortical thickness in the left dorsolateral prefrontal cortex (DLPFC), primary motor cortex, and anterior insula—changes that correlated directly with reductions in pain and physical disability. Conversely, brain morphology can also exhibit rapid plasticity in response to specific pharmacological interventions. For instance, short-term opioid administration in CLBP patients was shown to induce volumetric changes in reward- and affect-processing circuitry, such as the amygdala and inferior frontal gyrus, with some alterations persisting even after drug cessation (Younger et al., 2011). These divergent findings suggest that post-treatment cortical changes are dynamic and may depend heavily on the specific therapeutic modality and clinical outcome. Currently, it is unclear whether the specific brain structural abnormalities in CNSP patients can be restored following minimally invasive intervention. Furthermore, clinical outcomes for CNSP treatments are often heterogeneous; while many patients experience relief, a subset remains refractory to treatment (Li et al., 2014). This variability highlights the urgent need for objective neural markers to stratify patients. Recent research supports the clinical utility of this approach; for instance, a study integrating neuroimaging data with clinical metrics successfully identified distinct migraine subgroups that exhibited significantly different response rates to electroacupuncture (18% vs. 44%), thereby providing a concrete intermediate marker for treatment selection (Liu et al., 2023). Identifying similar baseline neuroanatomical features in CNSP could accelerate the development of neurophysiological subtypes, moving beyond a “one-size-fits-all” model toward personalized pain management.

To address these knowledge gaps, this study employed an exploratory surface-based morphometry (SBM) (Argall et al., 2006) approach to investigate whole-brain cortical alterations. We recruited a cohort of CNSP patients undergoing minimally invasive interventional treatments and acquired high-resolution magnetic resonance data at baseline and at a 3-month follow-up. The specific aims of this study were twofold: (1) to determine whether baseline cortical structure patterns can serve as prognostic markers to predict clinical treatment outcomes; and (2) to examine the longitudinal plasticity of the cerebral cortex following pain relief. By characterizing these morphological changes without *a priori* regional assumptions, we aim to provide robust longitudinal evidence clarifying the central mechanisms of CNSP recovery.

## 2 Methods

This prospective observational study was conducted at the Affiliated Hospital of North Sichuan Medical College. The Ethics Committee of the Affiliated Hospital of North Sichuan Medical College granted approval for the procedures (Approval No. 2023ER95-1), and all procedures were carried out in full compliance with the principles outlined in the Declaration of Helsinki. Written informed consent was obtained from all participants. They were thoroughly informed about the study prior to their participation.

### 2.1 Participants

CNSP Group: The individuals in the CNSP group were diagnosed with CNSP by two seasoned pain specialists from the Affiliated Hospital of North Sichuan Medical College. The diagnosis followed the chronic pain guidelines specified in the 11th edition of the International Classification of Diseases (ICD-11) (Scholz et al., 2019). Inclusion criteria: (1) Chronic pain in the neck and shoulders, which may extend to one or both arms, or may remain localized; (2) Duration of symptoms for at least 3 months. (3) Degenerative changes in the cervical spine identified through imaging techniques such as MRI or X-ray. (4) Patients who are either resistant to pharmacological treatment or experience significant adverse drug reactions and were already scheduled to undergo minimally invasive interventional therapy (Mao-Jiang et al., 2025) for cervical intervertebral disc at the Pain Medicine Department of our hospital as part of their routine clinical care, independent of this study; (5) No significant pain in other areas of the body; (6) Participants aged between 20 and 70 years, all of whom are right-handed; and (7) No medical conditions that would contraindicate MRI scanning. Exclusion criteria: (1) Presence of serious intracranial conditions, such as large-scale cerebral infarction, encephalomalacia, or brain tumors; (2) History of primary psychiatric disorders, including but not limited to anxiety, depression, Alzheimer’s disease, schizophrenia, or other mental or neurological disorders; and (3) Existence of severe systemic diseases, such as advanced cardiac, liver, or kidney failure.

HCs Group: Inclusion criteria: (1) Matched to the CNSP group in terms of age and handedness; (2) No contraindications for MRI; and (3) Absence of any acute or chronic pain symptoms. Exclusion criteria: (1) Presence of significant intracranial abnormalities; and (2) History of neurological or psychiatric disorders.

### 2.2 Clinical indicators assessment

Clinical indicators for all patients were assessed within 1 hour prior to each of the two MRI scans. The average pain intensity experienced over the past week was evaluated using the Visual Analogue Scale (Aitken, 1969) (VAS). This scale ranges from 0, indicating no pain, to 10, representing the most severe pain imaginable. Pain duration was defined as the time interval between the initial diagnosis of CNSP and the date of the preoperative brain MRI.

### 2.3 Minimally invasive interventional treatment procedures

Minimally invasive interventional treatment utilized a high-precision, CT-guided transforaminal injection technique to target the C3/4 and C5/6 cervical intervertebral foramina, selected for its ability to deliver therapeutic agents directly to the nerve roots while minimizing surgical trauma (Mao-Jiang et al., 2025). Following a 6 h fast, patients were positioned supine with the head rotated contralaterally, and a 64-row spiral CT (Philips) with 1.0 mm slice thickness was employed to map the anterolateral cervical approach. Under local anesthesia (1% lidocaine), a 14-cm, 22G coaxial trocar (Hakko) was advanced to the superior articular process and into the foramen under real-time imaging guidance. Upon confirming needle placement, a mixture of 10 mL ozone, 10 mL saline, and 1 mL iodixanol was injected; the contrast medium facilitated immediate CT verification of distribution within the spinal canal and around the nerve roots. Postoperative care included intravenous rehydration (500 mL sodium chloride) and a 3-day course of oral Erecoxib (0.1 g, b.i.d.) to manage inflammation and ensure patient recovery.

### 2.4 Imaging acquisition

All MRI scans were performed using a Siemens MAGNETOM Skyra 3.0 T MRI scanner, equipped with a standard 20-channel head–neck combined coil. CNSP patients underwent two MRI scans in total: one within 24 h prior to treatment and another 3 months after the minimally invasive intervention. The participants were placed in a horizontal position on the scanning table, with their heads secured by foam supports to minimize movement. To block out external sounds, earplugs were given. They were instructed to stay as motionless as possible throughout the imaging procedure.

Three-dimensional structural T1-weighted images were acquired utilizing a Magnetization-Prepared Rapid Acquisition with Gradient Echo (MP-RAGE) protocol. The imaging parameters included a repetition time (TR) of 2,240 ms, echo time (TE) of 3.73 ms, inversion time (TI) of 1,130 ms, flip angle of 9°, and a field of view (FOV) of 256 × 256 mm, with a slice thickness of 1 mm (no gap) and a total of 192 slices. The voxel dimensions were 1.0 × 1.0 × 1.0 mm.

### 2.5 Data preprocessing

Prior to the preprocessing pipeline, a quantitative quality control procedure was conducted using the Image Quality Rating (IQR) (Rosen et al., 2018) estimated by CAT12. The IQR serves as a comprehensive index of image quality, incorporating noise, inhomogeneity, and resolution parameters. To ensure data reliability, subjects with an IQR score lower than 75% (indicating below “satisfactory” quality) were excluded from the study. All neuroimaging MR data were processed and analyzed with the Computational Anatomy Toolbox (Gaser et al., 2024) (CAT12, https://neuro-jena.github.io/cat/), running in MATLAB 2022b. The preprocessing pipeline included bias-field correction, image segmentation, transformation into MNI space and Diffeomorphic Anatomical Registration Through Exponentiated Lie algebra (Ashburner, 2007) (DARTEL) normalization. Specifically, bias-field correction was first applied to address image intensity inhomogeneities (Banerjee and Maji, 2015). Then, tissue segmentation was performed using Tissue Probability Maps (TPMs) to separate gray matter, white matter, and cerebrospinal fluid (Ashburner and Friston, 2005). After segmentation, the images were registered to the East Asian brains space template (ICBM-152 brain template) using affine linear transformation (Mazziotta et al., 2001). Finally, spatial normalization was performed using the DARTEL algorithm to ensure precise alignment of anatomical structures across different individuals or time points (Ashburner, 2007).

### 2.6 Surface-based morphometry

Surface-based morphometry uses vertices instead of voxels to explicitly capture aspects of the brain’s surface, in particular the shape of its sulci and gyri. In this study, all four surface-based morphometric metrics (CT, GI, SD, and FD) were extracted using the CAT12 toolbox. Four different surface-based morphometry measures were used. (1) The estimation of Cortical Thickness (CT) and cortical surface reconstruction is performed using the Projection-Based Thickness (PBT) method. PBT estimates CT by focusing on the distance between gray matter and white matter voxels. This approach effectively mitigates partial volume effects, sulcal blurring, and sulcal asymmetry, thereby enhancing the precision of surface reconstruction (Dahnke et al., 2013). (2) The Gyrification Index (GI) describes the ratio of absolute mean curvature of the cortical surface to its perimeter, providing a compact quantification of folding complexity. In CAT12, GI is computed as a series of local GI values, allowing for a detailed analysis of cortical folding across the entire brain surface, instead of relying on a single global GI (Luders et al., 2006). (3) Sulcal Depth (SD) measures the straight-line distance between the central surface and the outer hull, providing insights into the complexity and variability of cortical surface morphology (Dahnke et al., 2013). (4) Fractal Dimension (FD) is an intrinsic measure of cortical complexity, based on the brain’s fractal nature, which consists of self-similar components at different scales. FD offers a compact and reliable assessment of cortical complexity, making it valuable for within-subject and inter-subject comparisons, as well as across different populations (Yotter et al., 2011).

### 2.7 Statistical analysis

The Shapiro–Wilk test was employed to assess the normality of the data distribution. Independent samples *t*-tests were conducted to compare continuous demographic variables between groups, while Chi-square tests were used for gender comparisons.

**Figure 1:**
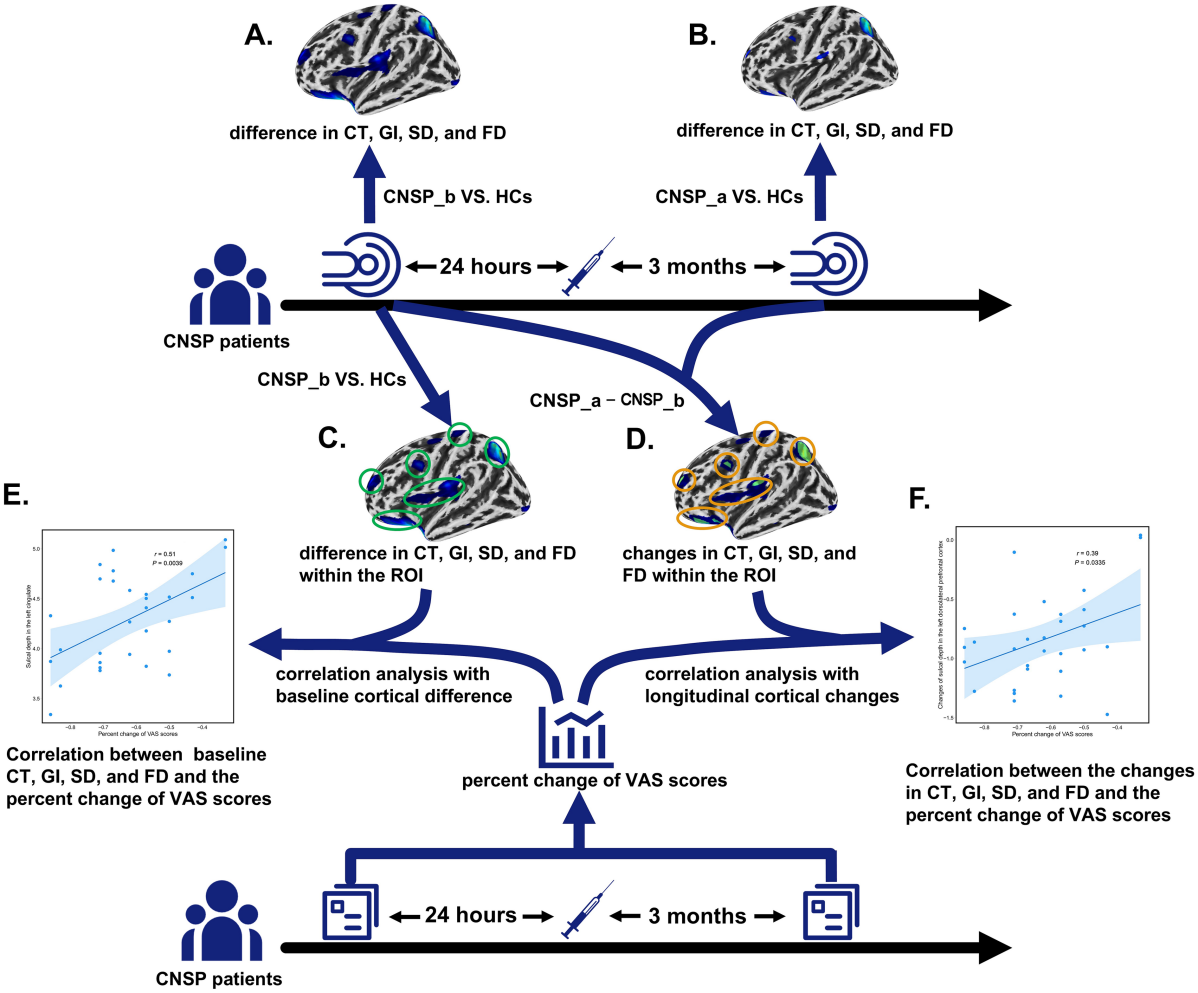
Flowchart illustrating our approach. **(A)** Comparison of inter-group differences in cortical thickness (CT), gyrification index (GI), sulcal depth (SD), and fractal dimension (FD) between the baseline chronic neck and shoulder pain (CNSP) group and the healthy control group. **(B)** Comparison of inter-group differences in CT, GI, SD, and FD between the CNSP group 3 months post-minimally invasive intervention and the healthy control group. **(C)** Identification of regions of interest (ROIs). Blue areas indicate brain regions exhibiting significant morphological differences compared to HCs at baseline. Green circles highlight these regions, which were defined as ROIs for subsequent analysis. **(D)** Longitudinal changes in ROIs after treatment. Green areas represent regions with persistent abnormalities (still significantly different from HCs), while blue areas represent regions showing recovery (no longer significantly different from HCs). **(E)** The correlation between the average value of each indicator in the ROI and the percentage change in visual analogue scale (VAS) score (calculated as the post-treatment VAS score at 3 months minus the baseline VAS score, divided by the baseline VAS score) is then analyzed. **(F)** The correlation between changes in CT, GI, SD, and FD (calculated as the average value of the indicator in the ROI at 3 months post-treatment minus the baseline value) and the percentage change in VAS score is analyzed. CNSP_b refers to CNSP patients before treatment, and CNSP_a refers to the same patients after treatment.

**Figure 2:**
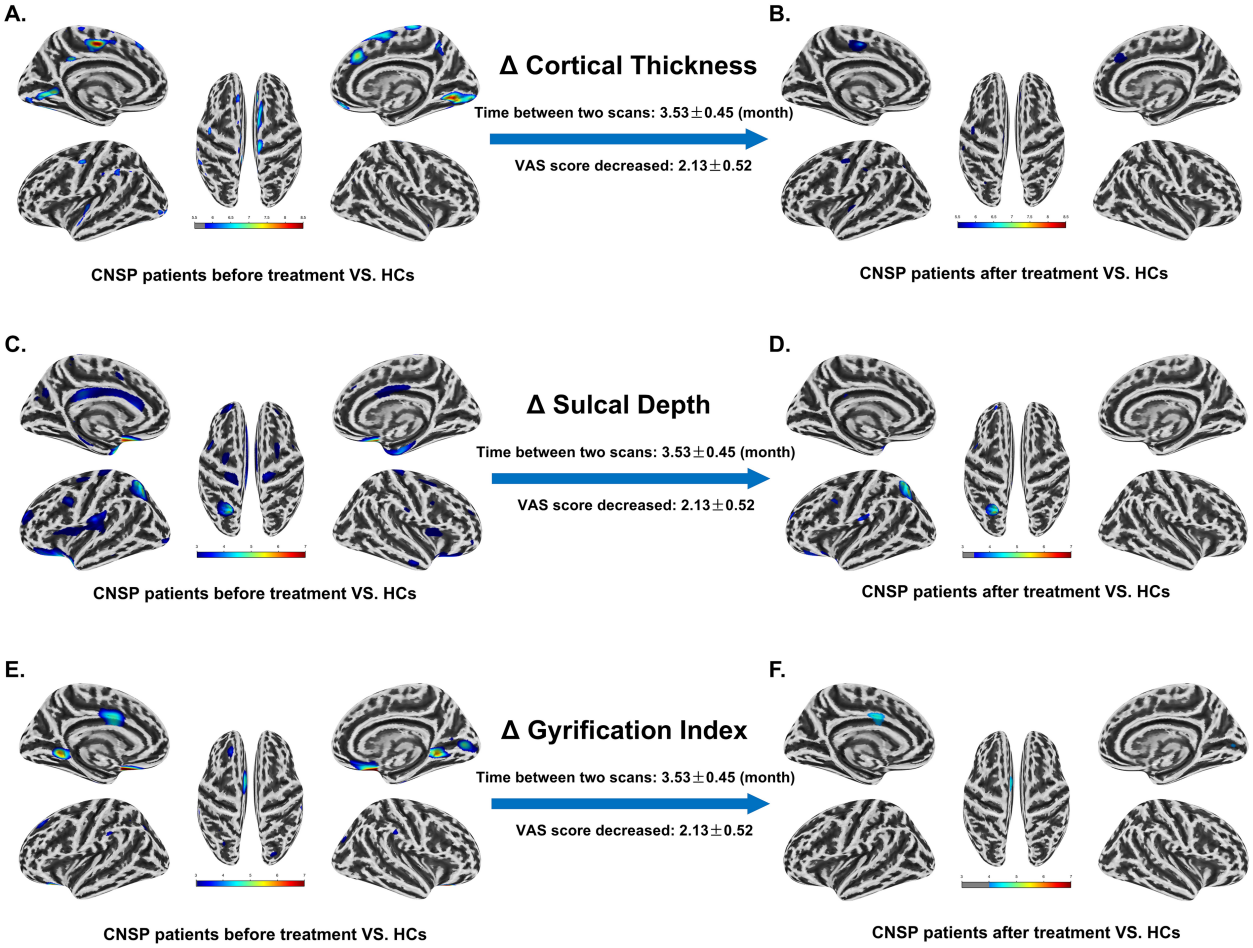
Abnormal changes and longitudinal variations in cortical features of chronic neck and shoulder pain (CNSP) patients compared to healthy controls (HCs) at baseline. **(A)** Differences in cortical thickness between CNSP patients and HCs at baseline. **(B)** Differences in cortical thickness between CNSP patients 3 months post-minimally invasive intervention and HCs. **(C)** Differences in cortical sulcal depth (SD) between CNSP patients and HCs at baseline. **(D)** Differences in cortical SD between CNSP patients 3 months post-minimally invasive intervention and HCs. **(E)** Differences in cortical gyrification index between CNSP patients and HCs at baseline. **(F)** Differences in cortical gyrification index between CNSP patients 3 months post-minimally invasive intervention and HCs. Clusters are significant at *p* < 0.05 (FDR-corrected), with an initial cluster-forming threshold of *p* < 0.001. The color bar indicates the voxel-wise *T*-value.

For the neuroimaging data, between-group differences in CT, GI, SD, and FD were evaluated using two-sample *t*-tests with age and gender included as covariates. Statistical inference was performed at the cluster level to control for multiple comparisons. An initial cluster-forming threshold of *p* < 0.001 was applied. Subsequently, clusters were considered significant if they survived False Discovery Rate (FDR) correction at the cluster level (*p* < 0.05). Two sets of inter-group comparisons were conducted: (1) baseline CNSP group vs. HCs (Figure 1A), and (2) CNSP group at 3 months post-minimally invasive intervention vs. HCs (Figure 1B).

Based on the neuroimaging results, brain regions showing significant differences between the baseline CNSP group and HCs were defined as regions of interest (ROIs) (Figure 1C). Partial correlation analyses were then performed to explore the associations between clinical improvements and brain metrics in CNSP patients, controlling for age, gender, and pain duration. Standard FDR correction was applied to correct for multiple testing across ROIs (*p* < 0.05). Two sets of partial correlation analyses were conducted:

Baseline Prediction: Correlation between the baseline mean values of each indicator within the ROIs and the percentage change in VAS scores (calculated as [post-treatment − baseline]/baseline) (Figure 1E).

Longitudinal Association: Correlation between the longitudinal changes in brain metrics (calculated as [3 months post-treatment − baseline] in the ROIs) (Figure 1D) and the percentage change in VAS scores (Figure 1F).

## 3 Results

### 3.1 Demographics and clinical measures

In this study, a total of 40 CNSP patients were initially recruited. However, seven patients were lost to follow-up after minimally invasive intervention (patients were unable to return to the hospital for subsequent studies due to various reasons), and thus failed to undergo the second MRI and clinical data collection 3 months after treatment. Additionally, 2 patients were excluded due to suboptimal image quality (CAT12 IQR < 75%) detected during the data quality assessment. As a result, the final sample consisted of 31 CNSP patients, and 30 HCs were also included. No significant differences were observed between the two groups in terms of age and gender (*p* > 0.05). The 31 CNSP patients included in the study showed a reduction in VAS scores from 6.18 ± 1.07 to 2.13 ± 0.52 after receiving minimally invasive intervention. Table 1 summarizes the demographic characteristics and behavioral measures for both the CNSP and control groups.

**Table 1:** Summary of demographic and behavioral characteristics.

Variables	CNSP (*n* = 31)	HCs (*n* = 30)	*P* value
Gender (male/female)	14/17	14/16	1
Age (years)	48.43 ± 7.15	46.34 ± 9.09	0.246
Duration of pain (months)	37.38 ± 16.48	–	–
VAS (baseline)	6.18 ± 1.07	–	–
VAS (after minimally invasive intervention therapy)	2.13 ± 0.52	–	–
Time interval between two scans (month)	3.53 ± 0.45	–	–

CNSP, chronic neck and shoulder pain; HCs, healthy controls; VAS, visual analogue scale.

### 3.2 Cortical thickness

At baseline, compared to the healthy control group, the CNSP group exhibited reduced CT in the left precentral gyrus, left lingual gyrus, left fusiform gyrus, left superior temporal gyrus, left supramarginal gyrus, left superior frontal gyrus, left paracentral lobule, right lingual gyrus, right superior frontal gyrus, right precentral gyrus, and right precuneus (*p* < 0.05, FDR corrected) (Table 2).

**Table 2:** Comparison of cortical thickness between patients with CNSP and healthy controls before and after minimally invasive intervention.

Brain region	Baseline				After minimally invasive intervention			
	Cluster size	*P* value (FDR)	Average thickness	Correlation with VAS (FDR)	Cluster size	*P* value (FDR corrected)	Average thickness	Correlation with VAS (longitudinal change, FDR)
Lingual Gyrus_L	240	<0.001	1.68 ± 0.14	/	/	/	1.76 ± 0.15	/
Paracentral Lobule_L	158.7	<0.001	2.35 ± 0.21	*r* = −0.44, 95% CI [−0.69, −0.10], *P* = 0.0152	103.5	0.013	2.42 ± 0.22	/
Superior Frontal Gyrus_L	151.1	0.024	1.92 ± 0.17	/	/	/	2.04 ± 0.18	/
Fusiform Gyrus_L	71	<0.001	2.14 ± 0.19	/	/	/	2.23 ± 0.20	/
Supramarginal Gyrus_L	69	0.016	1.55 ± 0.12	/	/	/	1.66 ± 0.13	/
Superior Temporal Gyrus_L	64.8	0.003	2.47 ± 0.23	/	/	/	2.52 ± 0.24	/
Precentral Gyrus_L	43	<0.001	1.71 ± 0.15	*r* = −0.57, 95% CI [−0.77, −0.26], *P* < 0.001	28	<0.001	1.81 ± 0.16	*r* = −0.54, 95% CI [−0.75, −0.22], *P* = 0.0021
Superior Frontal Gyrus_R	458.5	<0.001	1.64 ± 0.13	/	90	0.028	1.72 ± 0.14	/
Lingual Gyrus_R	392.8	0.019	2.38 ± 0.22	/	/	/	2.45 ± 0.23	/
Precuneus_R	111	0.006	1.97 ± 0.17	/	/	/	2.09 ± 0.18	/
Precentral Gyrus_R	40.8	0.011	1.59 ± 0.12	/	/	/	1.70 ± 0.13	/

Clusters are significant at *P* < 0.05, False Discovery Rate (FDR)-corrected, with an initial cluster-forming threshold of *P* < 0.001; CNSP, chronic neck and shoulder pain; VAS, visual analogue scale.

Three months following minimally invasive intervention, compared to the healthy control group, the CNSP group showed a reduction in CT in the left precentral gyrus, left paracentral lobule, and right superior frontal gyrus (*p* < 0.05, FDR corrected) (Table 2).

**Figure 3:**
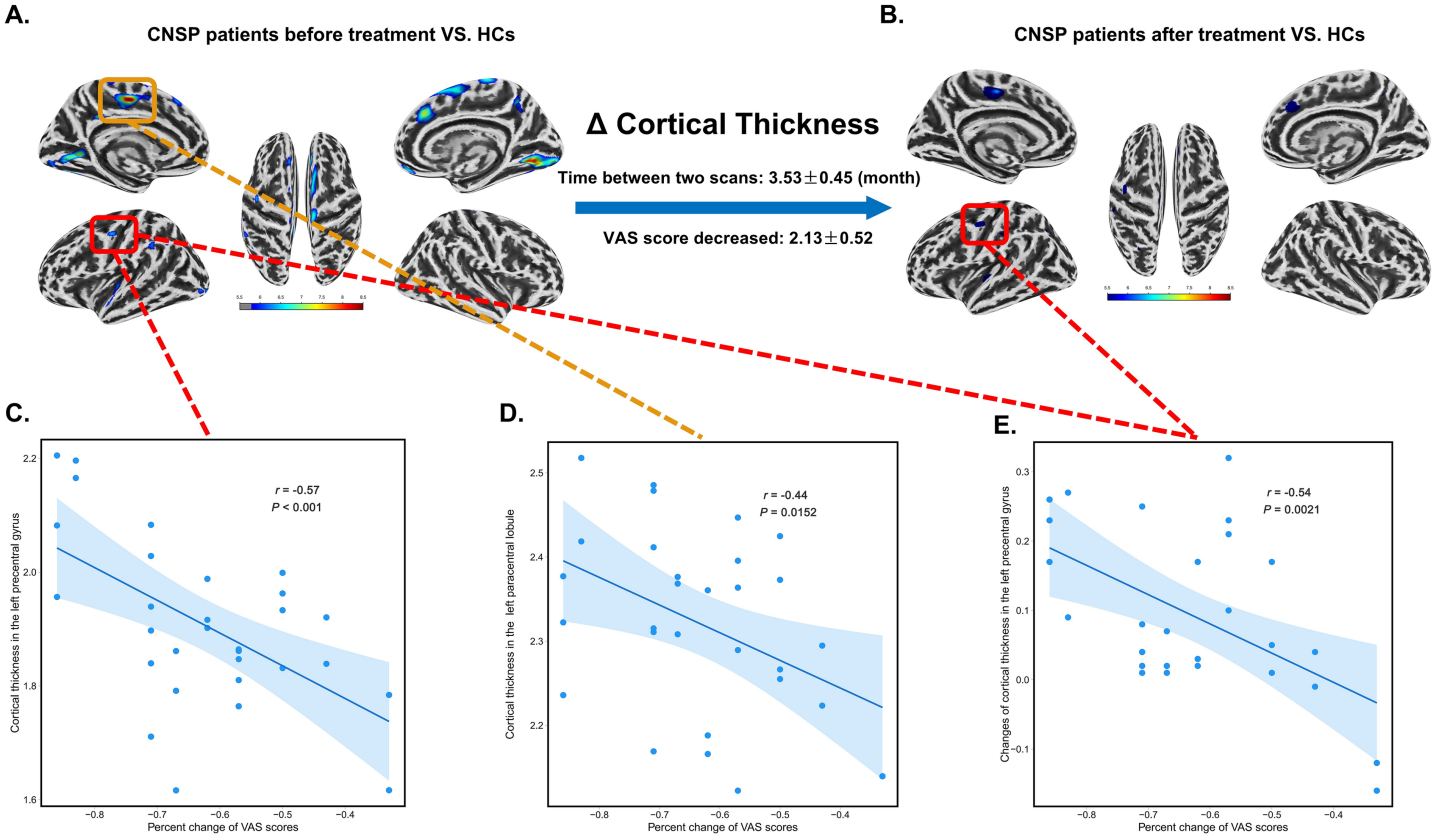
Partial correlation analysis between changes in cortical thickness (CT) and visual analogue scale (VAS) scores in chronic neck and shoulder pain (CNSP) patients. **(A)** Differences in CT between CNSP patients and healthy controls (HCs) at baseline. **(B)** Differences in CT between CNSP patients 3 months post-minimally invasive intervention and HCs. **(C)** Partial correlation analysis between CT of the left precentral gyrus at baseline in CNSP patients and the percent change in VAS scores. **(D)** Partial correlation analysis between CT of the left paracentral lobule at baseline in CNSP patients and the percent change in VAS scores. **(E)** Partial correlation analysis between changes in CT of the left precentral gyrus before and after minimally invasive intervention in CNSP patients and the percent change in VAS scores. Clusters are significant at *p* < 0.05 (FDR-corrected), with an initial cluster-forming threshold of *p* < 0.001. The color bar indicates the voxel-wise *T*-value.

As depicted in the Figures 2A,B, 3 months post-intervention, CNSP patients demonstrated a decrease in the regions exhibiting significant differences compared to baseline, with corresponding reductions in the *T*-values.

### 3.3 Sulcal depth

At baseline, compared with the healthy control group, the CNSP group showed deeper SD in the left lateral orbitofrontal cortex, left insular, left superior parietal lobule, left anterior cingulate cortex, left posterior cingulate cortex, left central sulcus, left dorsolateral prefrontal cortex, right lateral orbitofrontal cortex, right entorhinal cortex, right anterior cingulate cortex, right posterior cingulate cortex, right central sulcus, right insular, and right middle frontal gyrus (*p* < 0.05, FDR corrected) (Table 3).

**Table 3:** Comparison of sulcal depth between patients with CNSP and healthy controls before and after minimally invasive intervention.

Brain region	Baseline				After minimally invasive intervention			
	Cluster size	*P* value (FDR)	Average depth	Correlation with VAS (FDR)	Cluster size	*P* value (FDR)	Average depth	Correlation with VAS (longitudinal change, FDR)
Dorsolateral Prefrontal Cortex_L	503.5	<0.001	3.42 ± 0.26	/	124	0.003	3.32 ± 0.25	*r* = 0.39, 95% CI [0.03, 0.66], *P* = 0.0335
Superior Parietal Lobule_L	437.3	<0.001	7.15 ± 0.53	/	316.8	<0.001	6.88 ± 0.51	/
Insula_L	436.6	0.015	5.89 ± 0.44	/	33	0.021	5.74 ± 0.43	/
Central Sulcus_L	285.8	0.004	4.33 ± 0.31	*r* = 0.49, 95% CI [0.16, 0.72], *P* = 0.0062	/	/	4.13 ± 0.30	/
Posterior Cingulate Cortex_L	198.9	<0.001	5.26 ± 0.40	*r* = 0.51, 95% CI [0.18, 0.74], *P* = 0.0039	/	/	5.11 ± 0.39	*r* = 0.56, 95% CI [0.25, 0.77], *P* = 0.0014,
Anterior Cingulate Cortex_L	141.4	0.005	6.05 ± 0.48	/	/	/	5.83 ± 0.46	/
Lateral Orbitofrontal Cortex_L	110.8	0.013	5.57 ± 0.42	/	46	0.018	5.35 ± 0.40	/
Lateral Orbitofrontal Cortex_R	357	<0.001	4.12 ± 0.33	/	/	/	3.96 ± 0.31	/
Central Sulcus_R	193.9	0.046	6.93 ± 0.50	/	/	/	6.76 ± 0.49	/
Entorhinal Cortex_R	166.9	<0.001	3.67 ± 0.29	/	/	/	3.55 ± 0.28	/
Insula_R	152	0.028	5.04 ± 0.38	/	/	/	4.84 ± 0.36	/
Anterior Cingulate Cortex_R	150.4	0.003	7.49 ± 0.56	/	/	/	7.25 ± 0.54	/
Posterior Cingulate Cortex_R	148.7	0.003	7.36 ± 0.55	/	/	/	7.18 ± 0.53	/
Middle Frontal Gyrus_R	110.3	0.01	4.85 ± 0.36	/	/	/	4.69 ± 0.35	/

Clusters are significant at *P* < 0.05, False Discovery Rate (FDR)-corrected, with an initial cluster-forming threshold of *P* < 0.001; CNSP, chronic neck and shoulder pain; VAS, visual analogue scale.

Three months after minimally invasive intervention, compared to the healthy control group, the CNSP group exhibited deeper SD in the left superior parietal lobule, left lateral orbitofrontal cortex, left dorsolateral prefrontal cortex, and left insular (*p* < 0.05, FDR corrected) (Table 3).

As shown in the Figures 2C,D, 3 months post-intervention, CNSP patients exhibited a reduction in the regions with significant differences relative to baseline, and the corresponding *T*-values were also diminished.

### 3.4 Gyrification index

At baseline, compared to the healthy control group, the CNSP group demonstrated an increased GI in the left lateral orbitofrontal cortex, left anterior cingulate cortex, left posterior cingulate cortex, left lingual gyrus, left superior frontal gyrus, right medial orbitofrontal cortex, right lingual gyrus, and right parahippocampal gyrus (*p* < 0.05, FDR corrected) (Table 4).

**Table 4:** Comparison of gyrification index between patients with CNSP and healthy controls before and after minimally invasive intervention.

Brain region	Baseline				After minimally invasive intervention			
	Cluster size	*P* value (FDR)	Average gyrification	Correlation with VAS (FDR)	Cluster size	*P* value (FDR corrected)	Average gyrification	Correlation with VAS (longitudinal change, FDR)
Lateral Orbitofrontal Cortex_L	209.3	<0.001	27.84 ± 1.85	/	/	/	27.42 ± 1.81	/
Posterior Cingulate Cortex_L	203.7	<0.001	29.12 ± 2.14	/	95.7	0.018	28.68 ± 2.09	/
Lingual Gyrus_L	158.1	0.021	26.65 ± 1.28	/	/	/	26.25 ± 1.25	/
Anterior Cingulate Cortex_L	78.6	0.018	27.29 ± 1.63	/	/	/	26.88 ± 1.59	/
Superior Frontal Gyrus_L	77.9	<0.001	29.48 ± 2.36	/	/	/	29.04 ± 2.31	/
Medial Orbitofrontal Cortex_R	216.2	<0.001	29.03 ± 2.11	*r* = −0.49, 95% CI [−0.72, −0.16], *P* = 0.0061	/	/	28.59 ± 2.06	/
Lingual Gyrus_R	202.2	0.022	26.78 ± 1.39	/	/	/	26.38 ± 1.35	/
Parahippocampal Gyrus_R	40.9	0.019	28.89 ± 2.16	/	12.5	0.043	28.46 ± 2.11	/

Clusters are significant at *P* < 0.05, false discovery rate (FDR)-corrected, with an initial cluster-forming threshold of *P* < 0.001; CNSP, chronic neck and shoulder pain; VAS, visual analogue scale.

Three months after minimally invasive intervention, compared to the healthy control group, the CNSP group showed an increased GI in the left posterior cingulate cortex and right parahippocampal gyrus (*p* < 0.05, FDR corrected) (Table 4).

As observed in the figure, 3 months post-intervention, CNSP patients exhibited a reduction in the regions with significant differences compared to baseline, with corresponding decreases in the *T*-values (Figures 2E,F).

### 3.5 Fractal dimension

At baseline, there was no significant difference in the FD between the CNSP group and the healthy control group (*p* > 0.05, FDR corrected). Similarly, 3 months after minimally invasive intervention, no significant difference in the FD was found between the CNSP group and the healthy control group (*p* > 0.05, FDR corrected).

### 3.6 Results of partial correlation analysis

At baseline, the CT of the left precentral gyrus (*r* = −0.57, 95% CI [−0.77, −0.26], *p* < 0.001, FDR corrected) and the left paracentral lobule (*r* = −0.44, 95% CI [−0.69, −0.10], *p* = 0.0152, FDR corrected) in CNSP patients was negatively correlated with the percent change of VAS scores. This indicates that patients with a thicker cortex in these regions at baseline experienced greater pain relief (Figures 3A,C,D). Moreover, the changes of CT in the left precentral gyrus (*r* = −0.54, 95% CI [−0.75, −0.22], *p* = 0.0021, FDR corrected) before and after treatment were negatively correlated with the percent change of VAS scores, suggesting that a greater restoration (thickening) of the cortex was associated with a greater reduction in pain intensity (Figures 3A,B,E).

**Figure 4:**
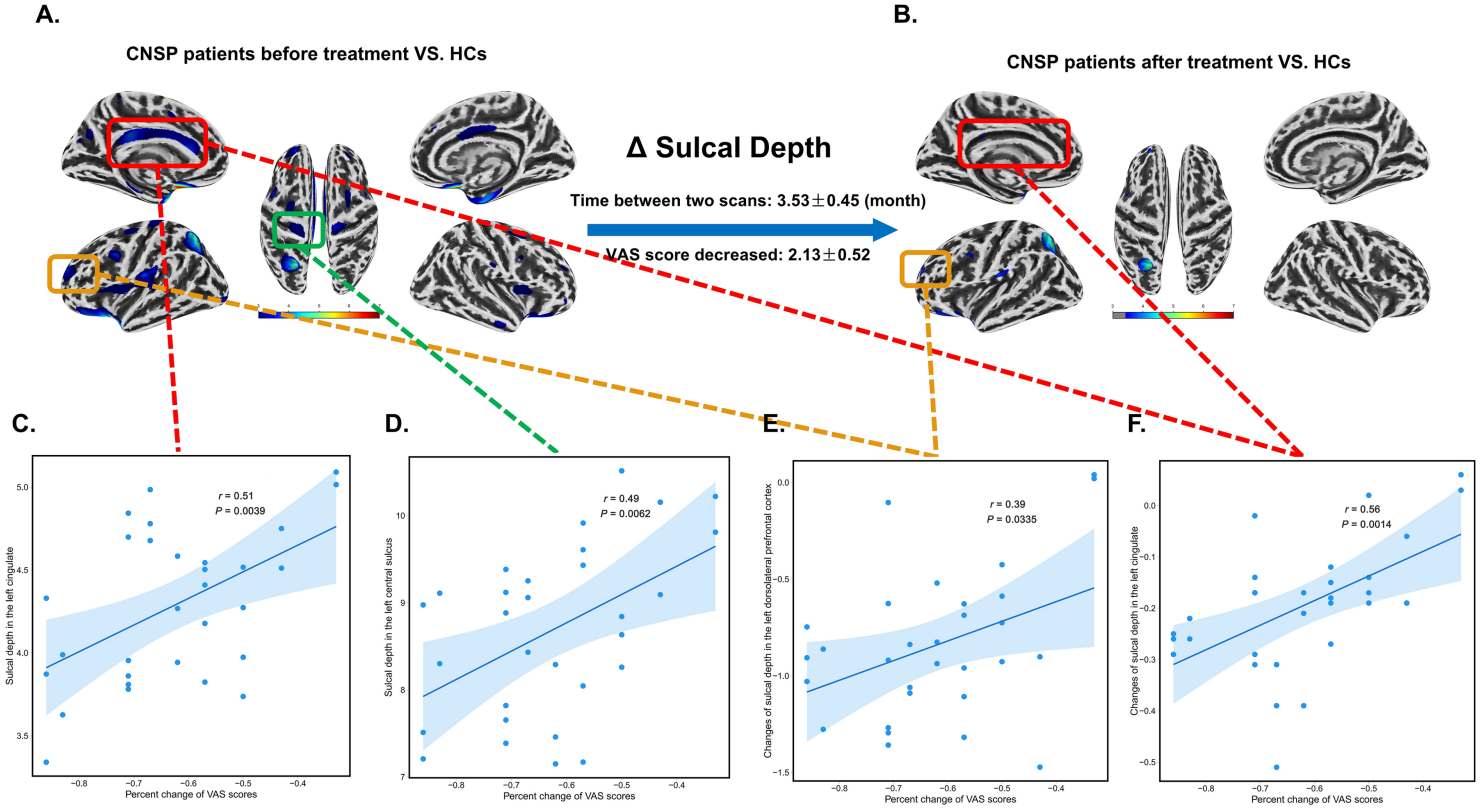
Partial correlation analysis between changes in sulcal depth (SD) and visual analogue scale (VAS) scores in chronic neck and shoulder pain (CNSP) patients. **(A)** Differences in cortical SD between CNSP patients and healthy controls (HCs) at baseline. **(B)** Differences in cortical SD between CNSP patients 3 months post-minimally invasive intervention and HCs. **(C)** Partial correlation analysis between SD of the left cingulate cortex at baseline in CNSP patients and the percent change in VAS scores. **(D)** Partial correlation analysis between SD of the left central sulcus at baseline in CNSP patients and the percent change in VAS scores. **(E)** Partial correlation analysis between changes in SD of the left dorsolateral prefrontal cortex before and after minimally invasive intervention in CNSP patients and the percent change in VAS scores. **(F)** Partial correlation analysis between changes in SD of the left cingulate cortex before and after minimally invasive intervention in CNSP patients and the percent change in VAS scores. Clusters are significant at *p* < 0.05 (FDR-corrected), with an initial cluster-forming threshold of *p* < 0.001. The color bar indicates the voxel-wise *T*-value.

At baseline, the SD in the left cingulate cortex (*r* = 0.51, 95% CI [0.18, 0.74], *p* = 0.0039, FDR corrected) and the left central sulcus (*r* = 0.49, 95% CI [0.16, 0.72], *p* = 0.0062, FDR corrected) of CNSP patients was positively correlated with the percent change of VAS scores, indicating that patients with deeper sulci in these areas at baseline tended to have poorer treatment outcomes (less pain relief) (Figures 4A,C,D). Furthermore, the changes of SD in the left dorsolateral prefrontal cortex (*r* = 0.39, 95% CI [0.03, 0.66], *p* = 0.0335, FDR corrected) and left cingulate cortex (*r* = 0.56, 95% CI [0.25, 0.77], *p* = 0.0014, FDR corrected) before and after treatment were positively correlated with the percent change of VAS scores. This positive correlation indicates that a better SD structural normalization was associated with greater pain relief (Figures 4A,B,E,F).

**Figure 5:**
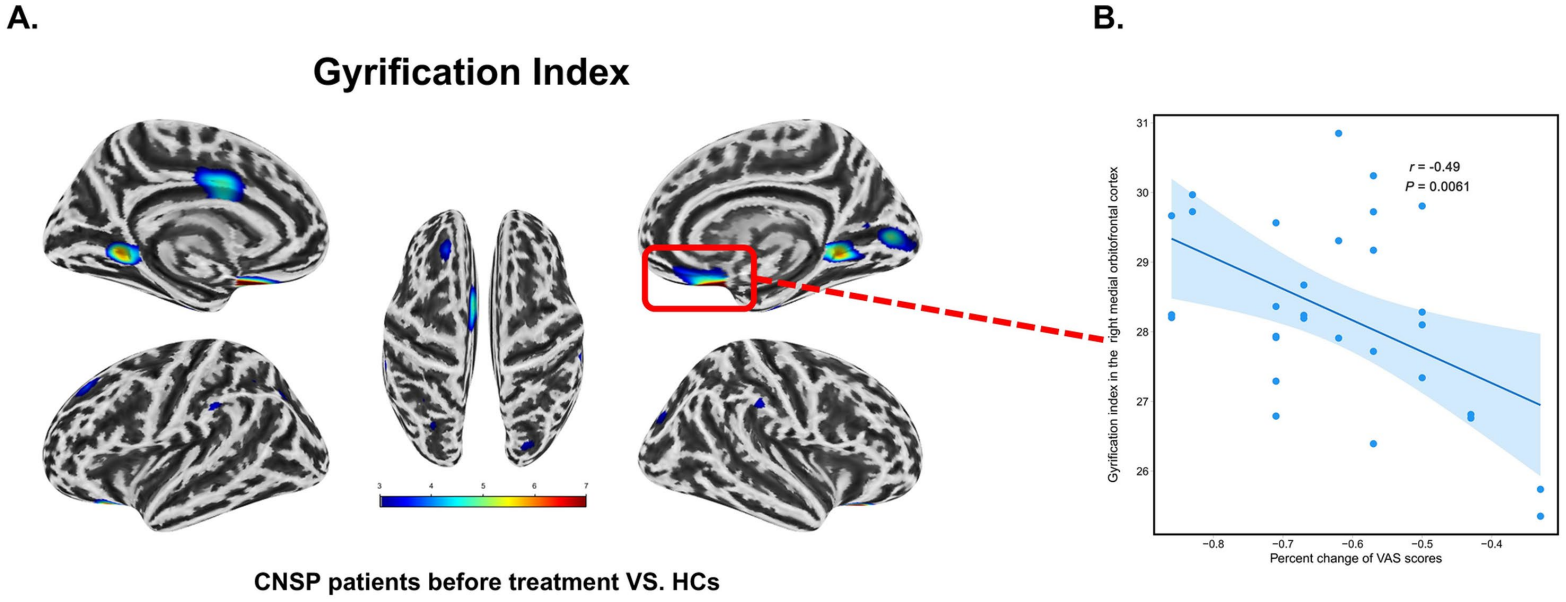
Partial correlation analysis between changes in gyrification index (GI) and visual analogue scale (VAS) scores in chronic neck and shoulder pain (CNSP) patients. **(A)** Differences in cortical GI between CNSP patients and healthy controls at baseline. **(B)** Partial correlation analysis between the GI of the right medial orbitofrontal cortex at baseline in CNSP patients and the percent change in VAS scores. Clusters are significant at *p* < 0.05 (FDR-corrected), with an initial cluster-forming threshold of *p* < 0.001. The color bar indicates the voxel-wise *T*-value.

At baseline, the GI of the right medial orbitofrontal cortex (*r* = −0.49, 95% CI [−0.72, −0.16], *p* = 0.0061, FDR corrected) in CNSP patients was negatively correlated with the percent change of VAS scores, suggesting that a higher gyrification index at baseline is a predictor of better treatment efficacy (greater pain relief) (Figures 5A,B).

## 4 Discussion

In the present study, we utilized surface-based morphometry (SBM) to investigate the longitudinal changes in cortical morphology (CT, GI, SD, and FD) in patients with CNSP before and 3 months after minimally invasive intervention. Our analysis yielded three main findings: (1) CNSP patients exhibited widespread cortical structural abnormalities at baseline, primarily involving regions associated with pain perception, emotional processing, and cognitive regulation; (2) minimal invasive intervention not only significantly alleviated pain but also reversed many of these cortical alterations, suggesting a trend towards structural normalization; and (3) specific baseline cortical features and their subsequent recovery were significantly correlated with clinical pain relief. Below, we interpret these widespread morphological changes by categorizing the affected regions according to their functional roles in the multidimensional experience of pain.

In this study, CNSP patients exhibited widespread cortical morphological alterations. To better understand the pathophysiological implications of these structural changes, we discussed the findings in the context of the functional roles of the affected regions. First, concerning the sensory-discriminative dimension of pain, we observed significant cortical thinning (reduced CT) and sulcal deepening (increased SD) in the bilateral central sulcus, paracentral lobule, and posterior cingulate cortex. These regions are fundamental components of the sensorimotor network, responsible for encoding the location and intensity of nociceptive stimuli (Jensen et al., 2023). We postulate that the structural alterations observed in these primary sensorimotor areas might be linked to activity-dependent plasticity driven by peripheral nociceptive input (Apkarian et al., 2004). Continuous nociceptive bombardment from the cervical spine leads to excessive neural activity in these projection areas. It is plausible that this chronic hyperactivity could potentially induce excitotoxicity or synaptic pruning, ultimately manifesting as neuronal loss and macroscopic gray matter atrophy (May, 2008). These results are partially consistent with previous findings of motor cortex atrophy in patients with chronic neck pain (Coppieters et al., 2018), supporting the hypothesis that the structural integrity of the sensorimotor cortex may be compromised by the persistent physiological demands of processing pain signals. Second, regarding pain emotional processing, we also observed a pattern of structural atrophy (reduced CT and/or increased SD) in key limbic and prefrontal regions, specifically the left anterior cingulate cortex (ACC), bilateral prefrontal cortex, and bilateral insula. The insula and ACC are core components of the “pain matrix,” crucial for integrating the affective-motivational dimension of pain (e.g., suffering) and interoceptive awareness (Bubb et al., 2018). The cortical thinning in these regions might reflect a deleterious consequence of chronic emotional stress. We speculate that the sustained engagement of these emotional hubs in processing negative affect could lead to neurodegenerative changes similar to those seen in the sensorimotor cortex. This interpretation aligns with previous studies showing gray matter loss in the ACC and insula in various chronic pain conditions (Apkarian et al., 2004), which is often associated with deficits in emotional regulation and increased pain unpleasantness (Valet et al., 2009). However, a distinct morphological pattern was observed in other regions associated with emotional and cognitive integration. Specifically, CNSP patients exhibited an increased Gyrification Index (GI) in the left lateral orbital frontal cortex (OFC), anterior cingulate cortex, posterior cingulate cortex, lingual gyrus, superior frontal gyrus, and the right medial orbital frontal cortex, lingual gyrus, and paracingulate cortex. Unlike the atrophy described above, higher gyrification typically reflects a more complex cortical folding pattern (Luders et al., 2006). We hypothesize that this increased complexity in these specific limbic and associative regions might represent a form of structural reorganization or adaptive plasticity (Fernández et al., 2016). Rather than definitive compensation, we cautiously interpret this phenomenon as a potential response to the complex demands of chronic pain. It is possible that the brain attempts to reorganize its local connectivity in these high-order processing areas to cope with the persistent emotional and cognitive load, resulting in increased cortical folding (Striedter et al., 2015; Klyachko and Stevens, 2003), although the precise cellular mechanisms require further investigation. Finally, structural alterations were evident in regions critical for pain cognitive regulation, specifically the left dorsolateral prefrontal cortex (DLPFC) and bilateral precentral gyrus, which showed significant cortical thinning. The DLPFC is a key node in the central executive network and plays a pivotal role in the “top-down” inhibition of ascending pain pathways (Lorenz et al., 2003). Structural deficits in the DLPFC are widely reported in chronic pain pathology (Apkarian et al., 2004). The cortical thinning observed in our study may potentially reflect a maladaptive response to the sustained demand for cognitive pain control. While the mechanism remains to be elucidated, this finding is consistent with meta-analyses demonstrating DLPFC gray matter reduction across various chronic pain conditions (Smallwood et al., 2013), reinforcing the link between executive control networks and the maintenance of chronic pain states.

Regarding the longitudinal changes, our results demonstrated that 3 months after minimally invasive intervention, the significant alleviation of pain was accompanied by a widespread normalization of cortical morphology (CT, SD, and GI) across the previously identified abnormal regions. This observation of structural reversibility is clinically significant, as it suggests that the baseline cortical alterations likely represented a state of maladaptive neuroplasticity driven by chronic nociceptive input, rather than permanent neurodegeneration (Calderone et al., 2024; Marzola et al., 2023). We suggest that this restoration may be attributed to the brain’s intrinsic capacity for reorganization. Following the intervention, the reduction in nociceptive signals likely alleviated the “sensory load” on pain-related networks was reduced, allowing neural circuits to re-establish their structural homeostasis (Tataranu and Rizea, 2025). Similar patterns of treatment-induced structural changes have been documented in other chronic conditions, such as anxiety (Steiger et al., 2017), major depression (Xu et al., 2019), and chronic pancreatitis (Muthulingam et al., 2018), highlighting the potential of effective pain management to reverse maladaptive cortical remodeling.

While the longitudinal results highlight the potential for structural recovery, it is equally important to understand how individual differences in baseline cortical structure influence clinical outcomes. Specifically, we found that higher baseline CT in the precentral gyrus and paracentral lobule, as well as lower baseline SD (shallower sulci) in the cingulate cortex and central sulcus, were significantly correlated with greater pain relief (more negative VAS percent change) after treatment. These findings suggest a clear link between pre-treatment structural integrity and clinical outcomes. As discussed above, these sensorimotor and limbic regions are vulnerable to atrophy driven by chronic nociceptive input. Patients with relatively preserved cortical thickness and sulcal depth in these critical nodes may possess a higher “neural reserve” (Stern, 2009). This structural integrity likely reflects a more resilient neural network capable of effective sensory-motor integration and emotional modulation. Consequently, patients with “less damaged” cortical substrates may be more responsive to minimally invasive interventions, leading to more pronounced pain relief (Virtanen et al., 2014). Furthermore, a significant correlation was observed between higher baseline GI in the right medial orbitofrontal cortex (mOFC) and better treatment outcomes. Consistent with our hypothesis that increased gyrification represents an adaptive structural reorganization, a higher GI in the mOFC implies a more robust compensatory mechanism for emotional regulation. Since the mOFC is pivotal for context-dependent pain modulation and coping strategies (Roy et al., 2012), patients who have successfully developed this structural complexity may possess enhanced cognitive-emotional resources to process pain (Kringelbach, 2005). Therefore, a higher degree of adaptive gyrification at baseline may prime the brain to benefit more effectively from the reduction of peripheral nociceptive drive achieved by the treatment.

Finally, beyond baseline predictors, we further examined the dynamic relationship between symptomatic improvement and structural plasticity. We found that the magnitude of pain relief (VAS percent change) was significantly correlated with the extent of structural reversal—specifically, changes in cortical thickness in the left precentral gyrus and changes in SD in the left dorsolateral prefrontal cortex (DLPFC) and cingulate cortex. This synchronization might be attributed to use-dependent neuroplasticity (Bliss et al., 2016). As the therapeutic intervention effectively alleviates the chronic nociceptive burden, the maladaptive processing load in these sensorimotor and cognitive control hubs is reduced, thereby promoting structural normalization. Consequently, patients experiencing greater pain relief exhibited more pronounced structural restoration. This parallel trajectory between clinical efficacy and cortical remodeling mirrors findings in other successful therapeutic interventions, suggesting that the observed morphological recovery is indeed functionally relevant (Sartorius et al., 2016; Zhou et al., 2022).

## 5 Limitations

In this study, although the minimally invasive interventions for CNSP patients were performed by a single surgeon using the same treatment method, and the analysis controlled for patients’ age, gender, and disease duration, the treatment efficacy may still be influenced by other uncontrollable factors [such as the degree of cervical disc herniation, spinal nerve root inflammation, postoperative care, etc. (Moustafa et al., 2024)]. Most importantly, the current study design lacked a control group receiving conservative therapy or sham intervention. Therefore, we cannot definitively attribute the observed cortical changes solely to the specific effects of the minimally invasive procedure, nor can we fully rule out placebo effects or the natural history of the disease. The interpretation of these changes as “recovery” or “adaptation” should be considered with caution. Furthermore, while we observed structural alterations in brain regions implicated in emotional processing (e.g., the anterior cingulate cortex and insula), the current study did not include specific behavioral assessments of anxiety or depression (such as the Self-Rating Anxiety Scale or Self-Rating Depression Scale). Chronic pain is frequently comorbid with emotional distress, which plays a crucial role in the “pain matrix (Bushnell et al., 2013).” The lack of these psychometric data prevents us from directly analyzing the correlation between the observed cortical changes in limbic regions and the patients’ emotional states. Future studies should include comprehensive psychological evaluations to better disentangle the specific contributions of sensory pain versus pain-related affect to cortical remodeling. Finally, it should be noted that the regions of interest (ROIs) used for the correlation analyses were defined based on the significant group differences observed at baseline. This analytical strategy introduces a circularity bias, often referred to as “double dipping,” which may potentially inflate the statistical strength of the observed correlations (Kriegeskorte et al., 2009). Ideally, this should be addressed using independent validation datasets or split-half cross-validation (Varoquaux et al., 2017). However, due to the limited sample size in the current study, such validation methods were not feasible without compromising statistical power. Therefore, the correlation results presented here should be interpreted as exploratory, and these findings require replication in future studies using larger, independent cohorts to confirm the robustness of the brain-behavior associations.

## 6 Conclusion

This study employed the surface-based morphometry method to analyze the MRI and clinical data of CNSP patients at baseline and 3 months after minimally invasive intervention, finding that CNSP patients exhibited abnormal cortical morphological changes in multiple brain regions associated with pain perception, emotional processing, and cognitive regulation, with some restoration observed after treatment. Furthermore, it was found that at baseline, the CT of the left precentral gyrus and left paracentral lobule, the SD of the left cingulate cortex and left central sulcus, and the GI of the right medial orbital frontal lobe were significantly correlated with the percent change of VAS scores. Changes in CT of the left precentral gyrus and changes in SD of the left cingulate cortex and left dorsolateral prefrontal cortex before and after the minimally invasive intervention were significantly correlated with the percent change of VAS scores. These findings provide valuable longitudinal data for understanding the central mechanisms of pain in CNSP patients and lay the foundation for individualized treatment based on cortical morphological changes in CNSP patients.

## Data Availability

The raw data supporting the conclusions of this article will be made available by the authors, without undue reservation.

## References

[ref1] Aitken R. C. (1969). Measurement of feelings using visual analogue scales. Proc. R. Soc. Med. 62, 989–993. 4899510 10.1177/003591576906201005 PMC1810824

[ref2] Apkarian A. V. Sosa Y. Sonty S. Levy R. M. Harden R. N. Parrish T. B. . (2004). Chronic back pain is associated with decreased prefrontal and thalamic gray matter density. J. Neurosci. 24, 10410–10415. doi: 10.1523/jneurosci.2541-04.2004, 15548656 PMC6730296

[ref3] Argall B. D. Saad Z. S. Beauchamp M. S. (2006). Simplified intersubject averaging on the cortical surface using SUMA. Hum. Brain Mapp. 27, 14–27. doi: 10.1002/hbm.20158 16035046 PMC6871368

[ref4] Ashburner J. (2007). A fast diffeomorphic image registration algorithm. Neuroimage 38, 95–113. doi: 10.1016/j.neuroimage.2007.07.007 17761438

[ref5] Ashburner J. Friston K. J. (2005). Unified segmentation. NeuroImage 26, 839–851. doi: 10.1016/j.neuroimage.2005.02.018, 15955494

[ref6] Banerjee A. Maji P. (2015). Rough sets and stomped normal distribution for simultaneous segmentation and bias field correction in brain MR images. IEEE Trans. Image Process. 24, 5764–5776. doi: 10.1109/TIP.2015.2488900, 26462197

[ref7] Bliss T. V. P. Collingridge G. L. Kaang B. Zhuo M. (2016). Synaptic plasticity in the anterior cingulate cortex in acute and chronic pain. Nat. Rev. Neurosci. 17, 485–496. doi: 10.1038/nrn.2016.68, 27307118

[ref8] Bubb E. J. Metzler-Baddeley C. Aggleton J. P. (2018). The cingulum bundle: anatomy, function, and dysfunction. Neurosci. Biobehav. Rev. 92, 104–127. doi: 10.1016/j.neubiorev.2018.05.008, 29753752 PMC6090091

[ref9] Bushnell M. C. Ceko M. Low L. A. (2013). Cognitive and emotional control of pain and its disruption in chronic pain. Nat. Rev. Neurosci. 14, 502–511. doi: 10.1038/nrn3516, 23719569 PMC4465351

[ref10] Calderone A. Latella D. Cardile D. Gangemi A. Corallo F. Rifici C. . (2024). The role of neuroinflammation in shaping neuroplasticity and recovery outcomes following traumatic brain injury: a systematic review. Int. J. Mol. Sci. 25:11708. doi: 10.3390/ijms252111708, 39519259 PMC11546226

[ref11] Cohen S. P. (2015). Epidemiology, diagnosis, and treatment of neck pain. Mayo Clin. Proc. 90, 284–299. doi: 10.1016/j.mayocp.2014.09.008, 25659245

[ref12] Coppieters I. De Pauw R. Caeyenberghs K. Lenoir D. DeBlaere K. Genbrugge E. . (2018). Differences in white matter structure and cortical thickness between patients with traumatic and idiopathic chronic neck pain: associations with cognition and pain modulation? Hum. Brain Mapp. 39, 1721–1742. doi: 10.1002/hbm.23947, 29327392 PMC6866349

[ref13] Dahnke R. Yotter R. A. Gaser C. (2013). Cortical thickness and central surface estimation. NeuroImage 65, 336–348. doi: 10.1016/j.neuroimage.2012.09.050, 23041529

[ref14] De Pauw R. Coppieters I. Caeyenberghs K. Kregel J. Aerts H. Lenoir D. . (2019). Associations between brain morphology and motor performance in chronic neck pain: a whole-brain surface-based morphometry approach. Hum. Brain Mapp. 40, 4266–4278. doi: 10.1002/hbm.24700, 31222905 PMC6865716

[ref15] Dieleman J. L. Cao J. Chapin A. Chen C. Li Z. Liu A. . (2020). US health care spending by payer and health condition, 1996-2016. JAMA 323, 863–884. doi: 10.1001/jama.2020.0734, 32125402 PMC7054840

[ref16] Fejer R. Kyvik K. O. Hartvigsen J. (2006). The prevalence of neck pain in the world population: a systematic critical review of the literature. Eur. Spine J. 15, 834–848. doi: 10.1007/s00586-004-0864-4, 15999284 PMC3489448

[ref17] Fernández V. Llinares-Benadero C. Borrell V. (2016). Cerebral cortex expansion and folding: what have we learned? EMBO J. 35, 1021–1044. doi: 10.15252/embj.201593701, 27056680 PMC4868950

[ref18] Gaser C. Dahnke R. Thompson P. M. Kurth F. Luders E. The Alzheimer's Disease Neuroimaging Initiative (2024). CAT: a computational anatomy toolbox for the analysis of structural MRI data. Gigascience 13:giae049. doi: 10.1093/gigascience/giae049 39102518 PMC11299546

[ref19] Jensen M. A. Huang H. Valencia G. O. Klassen B. T. van den Boom M. A. Kaufmann T. J. . (2023). A motor association area in the depths of the central sulcus. Nat. Neurosci. 26, 1165–1169. doi: 10.1038/s41593-023-01346-z, 37202552 PMC10322697

[ref20] Klyachko V. A. Stevens C. F. (2003). Connectivity optimization and the positioning of cortical areas. Proc. Natl. Acad. Sci. USA 100, 7937–7941. doi: 10.1073/pnas.0932745100, 12796510 PMC164691

[ref21] Kriegeskorte N. Simmons W. K. Bellgowan P. S. F. Baker C. I. (2009). Circular analysis in systems neuroscience: the dangers of double dipping. Nat. Neurosci. 12, 535–540. doi: 10.1038/nn.2303, 19396166 PMC2841687

[ref22] Kringelbach M. L. (2005). The human orbitofrontal cortex: linking reward to hedonic experience. Nat. Rev. Neurosci. 6, 691–702. doi: 10.1038/nrn1747, 16136173

[ref23] Li B. Xu X. X. Du Y. Yang H. F. Li Y. Zhang Q. . (2014). CT-guided chemonucleolysis combined with psoas compartment block in lumbar disc herniation: a randomized controlled study. Pain Med. 15, 1470–1476. doi: 10.1111/pme.12491 25041326

[ref24] Liu J. Quan S. Zhao L. Yuan K. Wang Y. Zhang Y. . (2023). Evaluation of a clustering approach to define distinct subgroups of patients with migraine to select Electroacupuncture treatments. Neurology 101, e699–e709. doi: 10.1212/WNL.0000000000207484, 37349112 PMC10437024

[ref25] Lorenz J. Minoshima S. Casey K. L. (2003). Keeping pain out of mind: the role of the dorsolateral prefrontal cortex in pain modulation. Brain 126, 1079–1091. doi: 10.1093/brain/awg102, 12690048

[ref26] Luders E. Thompson P. M. Narr K. L. Toga A. W. Jancke L. Gaser C. (2006). A curvature-based approach to estimate local gyrification on the cortical surface. NeuroImage 29, 1224–1230. doi: 10.1016/j.neuroimage.2005.08.049, 16223589

[ref27] Mao-Jiang Y. Xian Q. Hong-Ying Y. Han-Wen Z. Zhi-Qiang Q. Li-Bing H. . (2025). Hope for 17 patients with chronic cluster headache: efficacy evaluation of upper cervical spinal nerve root release surgery (2020-2023). Front. Neurol. 16:1662677. doi: 10.3389/fneur.2025.1662677, 41293412 PMC12640831

[ref28] Marzola P. Melzer T. Pavesi E. Gil-Mohapel J. Brocardo P. S. (2023). Exploring the role of neuroplasticity in development, aging, and neurodegeneration. Brain Sci. 13:1610. doi: 10.3390/brainsci13121610, 38137058 PMC10741468

[ref29] May A. (2008). Chronic pain may change the structure of the brain. Pain 137, 7–15. doi: 10.1016/j.pain.2008.02.034, 18410991

[ref30] Mazziotta J. Toga A. Evans A. Le Goualher G. Boomsma D. Cannon T. . (2001). A probabilistic atlas and reference system for the human brain: international consortium for brain mapping (ICBM). Philos. Trans. R. Soc. Lond. Ser. B Biol. Sci. 356, 1293–1322. doi: 10.1098/rstb.2001.0915, 11545704 PMC1088516

[ref31] Moustafa I. M. Ozsahin D. U. Mustapha M. T. Ahbouch A. Oakley P. A. Harrison D. E. (2024). Utilizing machine learning to predict post-treatment outcomes in chronic non-specific neck pain patients undergoing cervical extension traction. Sci. Rep. 14:11781. doi: 10.1038/s41598-024-62812-7, 38783089 PMC11116459

[ref32] Murray C. J. L. Atkinson C. Bhalla K. Birbeck G. Weisskopf R. Wulf S. . (2013). The state of US health, 1990-2010: burden of diseases, injuries, and risk factors. JAMA 310, 591–608. doi: 10.1001/jama.2013.13805 23842577 PMC5436627

[ref33] Muthulingam J. Olesen S. S. Hansen T. M. Seminowicz D. A. Burrowes S. Drewes A. M. . (2018). Progression of structural brain changes in patients with chronic pancreatitis and its association to chronic pain: a 7-year longitudinal follow-up study. Pancreas 47, 1267–1276. doi: 10.1097/MPA.0000000000001151, 30211804

[ref34] Niddam D. M. Lee S. Su Y. Chan R. (2019). Altered cortical morphology in patients with chronic shoulder pain. Neurosci. Lett. 712:134515. doi: 10.1016/j.neulet.2019.134515, 31560996

[ref35] Rosen A. F. G. Roalf D. R. Ruparel K. Blake J. Seelaus K. Villa L. P. . (2018). Quantitative assessment of structural image quality. NeuroImage 169, 407–418. doi: 10.1016/j.neuroimage.2017.12.059, 29278774 PMC5856621

[ref36] Roy M. Shohamy D. Wager T. D. (2012). Ventromedial prefrontal-subcortical systems and the generation of affective meaning. Trends Cogn. Sci. 16, 147–156. doi: 10.1016/j.tics.2012.01.005, 22310704 PMC3318966

[ref37] Sartorius A. Demirakca T. Böhringer A. Clemm von Hohenberg C. Aksay S. S. Bumb J. M. . (2016). Electroconvulsive therapy increases temporal gray matter volume and cortical thickness. Eur. Neuropsychopharmacol. 26, 506–517. doi: 10.1016/j.euroneuro.2015.12.036, 26792445

[ref38] Scholz J. Finnerup N. B. Attal N. Wang Q. Barke A. Rief W. . (2019). The IASP classification of chronic pain for ICD-11: chronic neuropathic pain. Pain 160, 53–59. doi: 10.1097/j.pain.0000000000001365, 30586071 PMC6310153

[ref39] Seminowicz D. A. Wideman T. H. Naso L. Hatami-Khoroushahi Z. Fallatah S. Ware M. A. . (2011). Effective treatment of chronic low back pain in humans reverses abnormal brain anatomy and function. J. Neurosci. 31, 7540–7550. doi: 10.1523/JNEUROSCI.5280-10.2011, 21593339 PMC6622603

[ref40] Smallwood R. F. Laird A. R. Ramage A. E. Parkinson A. L. Lewis J. Clauw D. J. . (2013). Structural brain anomalies and chronic pain: a quantitative meta-analysis of gray matter volume. J. Pain 14, 663–675. doi: 10.1016/j.jpain.2013.03.001, 23685185 PMC4827858

[ref41] Steiger V. R. Brühl A. B. Weidt S. Delsignore A. Rufer M. Jäncke L. . (2017). Pattern of structural brain changes in social anxiety disorder after cognitive behavioral group therapy: a longitudinal multimodal MRI study. Mol. Psychiatry 22, 1164–1171. doi: 10.1038/mp.2016.217, 27922605

[ref42] Stern Y. (2009). Cognitive reserve. Neuropsychologia 47, 2015–2028. doi: 10.1016/j.neuropsychologia.2009.03.004, 19467352 PMC2739591

[ref43] Striedter G. F. Srinivasan S. Monuki E. S. (2015). Cortical folding: when, where, how, and why? Annu. Rev. Neurosci. 38, 291–307. doi: 10.1146/annurev-neuro-071714-034128, 25897870

[ref44] Tataranu L. G. Rizea R. E. (2025). Neuroplasticity and nervous system recovery: cellular mechanisms, therapeutic advances, and future prospects. Brain Sci. 15:400. doi: 10.3390/brainsci15040400, 40309875 PMC12025631

[ref45] Valet M. Gündel H. Sprenger T. Sorg C. Mühlau M. Zimmer C. . (2009). Patients with pain disorder show gray-matter loss in pain-processing structures: a voxel-based morphometric study. Psychosom. Med. 71, 49–56. doi: 10.1097/PSY.0b013e31818d1e02, 19073757

[ref46] Varoquaux G. Raamana P. R. Engemann D. A. Hoyos-Idrobo A. Schwartz Y. Thirion B. (2017). Assessing and tuning brain decoders: cross-validation, caveats, and guidelines. NeuroImage 145, 166–179. doi: 10.1016/j.neuroimage.2016.10.038, 27989847

[ref47] Virtanen S. Utriainen K. T. Parkkola R. Airaksinen J. K. Laitio R. Scheinin H. . (2014). White matter damage of the brain is associated with poor outcome in vascular surgery patients with claudication: a pilot study. Eur. J. Vasc. Endovasc. Surg. 48, 687–693. doi: 10.1016/j.ejvs.2014.08.025, 25544158

[ref48] Xu J. Wang J. Bai T. Zhang X. Li T. Hu Q. . (2019). Electroconvulsive therapy induces cortical morphological alterations in major depressive disorder revealed with surface-based morphometry analysis. Int. J. Neural Syst. 29:1950005. doi: 10.1142/s0129065719500059, 31387489

[ref49] Yotter R. A. Nenadic I. Ziegler G. Thompson P. M. Gaser C. (2011). Local cortical surface complexity maps from spherical harmonic reconstructions. NeuroImage 56, 961–973. doi: 10.1016/j.neuroimage.2011.02.007, 21315159

[ref50] Younger J. W. Chu L. F. D'Arcy N. T. Trott K. E. Jastrzab L. E. Mackey S. C. (2011). Prescription opioid analgesics rapidly change the human brain. Pain 152, 1803–1810. doi: 10.1016/j.pain.2011.03.028, 21531077 PMC3138838

[ref51] Zhou X. Tan Y. Yu H. Liu J. Lan X. Deng Y. . (2022). Early alterations in cortical morphology after neoadjuvant chemotherapy in breast cancer patients: a longitudinal magnetic resonance imaging study. Hum. Brain Mapp. 43, 4513–4528. doi: 10.1002/hbm.25969, 35665982 PMC9491291

